# Comparison of endothelial cell attachment on surfaces of biodegradable polymer-coated magnesium alloys in a microfluidic environment

**DOI:** 10.1371/journal.pone.0205611

**Published:** 2018-10-10

**Authors:** Lumei Liu, Sang-Ho Ye, Xinzhu Gu, Teal Russell, Zhigang Xu, Jagannathan Sankar, William R. Wagner, Young-Choon Lee, Yeoheung Yun

**Affiliations:** 1 National Science Foundation-Engineering Research Center for Revolutionizing Metallic Biomaterials, North Carolina Agricultural and Technical State University, Greensboro, North Carolina, United States of America; 2 FIT BEST Laboratory, Department of Chemical, Biological, and Bioengineering, North Carolina Agricultural and Technical State University, Greensboro, North Carolina, United States of America; 3 McGowan Institute for Regenerative Medicine, University of Pittsburgh, Pittsburgh, Pennsylvania, United States of America; 4 Department of Surgery, University of Pittsburgh, Pittsburgh, Pennsylvania, United States of America; 5 Jeonbuk Technopark, Regional Industry Promotion Office, Chonbuk Province, Jeonju, South Korea; Hungarian Academy of Sciences, HUNGARY

## Abstract

Polymeric coatings can provide temporary stability to bioresorbable metallic stents at the initial stage of deployment by alleviating rapid degradation and providing better interaction with surrounding vasculature. To understand this interfacing biocompatibility, this study explored the endothelial-cytocompatibility of polymer-coated magnesium (Mg) alloys under static and dynamic conditions compared to that of non-coated Mg alloy surfaces. Poly (carbonate urethane) urea (PCUU) and poly (lactic-co-glycolic acid) (PLGA) were coated on Mg alloys (WE43, AZ31, ZWEKL, ZWEKC) and 316L stainless steel (316L SS, control sample), which were embedded into a microfluidic device to simulate a vascular environment with dynamic flow. The results from attachment and viability tests showed that more cells were attached on the polymer-coated Mg alloys than on non-coated Mg alloys in both static and dynamic conditions. In particular, the attachment and viability on PCUU-coated surfaces were significantly higher than that of PLGA-coated surfaces of WE43 and ZWEKC in both static and dynamic conditions, and of AZ31 in dynamic conditions (P<0.05). The elementary distribution map showed that there were relatively higher Carbon weight percentages and lower Mg weight percentages on PCUU-coated alloys than PLGA-coated alloys. Various levels of pittings were observed underneath the polymer coatings, and the pittings were more severe on the surface of Mg alloys that corroded rapidly. Polymer coatings are recommended to be applied on Mg alloys with relatively low corrosion rates, or after pre-stabilizing the substrate. PCUU-coating has more selective potential to enhance the biocompatibility and mitigate the endothelium damage of Mg alloy stenting.

## Introduction

Magnesium (Mg)-based alloys are bioresorbable scaffold materials that demonstrate enhanced properties of biodegradability and biocompatibility, and thus are being investigated for medical applications, such as atherosclerosis treatment [1, 2]. Magnesium-based alloys have been studied in both *in vitro* and animal models [3–5], and Mg-based metal scaffolds have been tested in clinical trials for cardiovascular atherosclerosis [6]. However, there are few studies on magnesium alloys for other atherosclerotic diseases, such as intracranial atherosclerotic disease (ICAD), which has been claimed to be the most common cause of stroke worldwide [7]. Current treatments of ICAD are brain stents, medications, and surgery [8]. Brain stents made of permanent metallic materials have some risks upon implantation: artery puncture, stent movement, damage to the lining of the vessel causing an artery dissection, bleeding into the brain, and stroke from artery blockage [9]. Based on the studies of Mg alloys for cardiovascular application, Mg alloys show promise in addressing these risks. Magnesium-based alloys are potentially biocompatible and provide high tensile strength (~542 MPa [10]) with lightweight properties, thus presenting new opportunities for cerebral stent application.

One of the limitations of using magnesium-based alloys is the uncontrollability of degradation *in vivo* due to the electrochemically active property of magnesium alloys. Biodegradable polymer coatings are used to provide temporary corrosion resistance to Mg-based alloys for both orthopedic and cardiovascular applications [11–14]; however, it has been shown that some durable polymer drug-eluting stents are associated with increased risk of late stent thrombosis, with evidence of incomplete endothelialization, delayed arterial healing [15–21]. The ideal polymer coating can minimize surgical damage to the artery, and promote tissue regeneration where any damage occurs. Applied degradable coatings can minimize the damage caused by stents, and even aid in the regeneration where damage has occurred. Poly(carbonate urethane) urea (PCUU) and poly (lactic-co-glycolic acid) (PLGA) have been studied as Mg-based alloy coatings, and have been reported previously [22]. PLGA has been evaluated as a coating on Mg-based alloys because of its biocompatibility. PLGA-coated Mg-based alloys have significantly decreased degradation rates and better compatibility with blood and osteoblast cells than non-coated Mg-based alloys [23, 24]. PCUU-coated Mg-based stents (AZ31, 3% Al, 1% Zn) have improved corrosion resistance and reduced thrombotic deposition *in vitro* compared to PLGA-coated and non-coated alloy stents [22]. The cellular response under dynamic conditions is rarely studied and the coatings on different Mg-based alloys might have different corrosion behaviors.

In this paper, endothelial cells are used to test cytocompatibility of polymer-coated Mg-based alloys in a vascular-mimetic shear stress environment. A microfluidic system was used as a test bed to simulate a physiological environment [3] with specific shear stress. The study is innovative because in the dynamic conditions, the responses of endothelial cells to polymer-coated Mg-based alloys are more representative of the *in vivo* environment compared to the test in static conditions. Observing the interaction of endothelial cells with coated surfaces using scanning electron microscopy (SEM) facilitates the screening of more compatible coatings and alloys. Endothelial cell attachment and morphology, including elementary distribution and pittings underneath the coatings, were characterized to study the effects of different coatings.

## Materials and methods

### Alloy preparation

Stainless steel (316L SS) was purchased from McMaster-Carr (Douglasville, GA). Selected magnesium-based alloys include casted WE43 (Magnesium Elektron North America Inc., Manchester, NJ), extruded AZ31 (Goodfellow, Oakdale, PA), ZWEKL and ZWEKC. ZWEK was fabricated with 99.97% Mg, 99.99% Zn and master alloys Mg-30%Y, Mg-30% Rare earth and Mg-30%Zr in NSF-funded ERC for Revolutionizing Metallic Biomaterials (RMB) at NC A&T State University according to the previous report [25]. ZWEK-L and -C samples were cut from the longitudinal and cross-sectional directions from the extruded rod, respectively. All alloys were cut and polished into cuboids of 5 mm in length, 2 mm in width, and 3 mm in thickness. The samples were mechanically polished with silicon carbide (SiC) paper progressively up to 1000 grit with water and then polished with 1200 grit SiC paper with isopropyl alcohol. Specimens were then ultrasonically cleaned sequentially with acetone and ethanol, and dried using compressed air. The grain structures of magnesium-based alloys were observed under SEM (S1A Fig). The percentages of secondary phases and impurity areas (S1B Fig) were analyzed by ImageJ software (US National Institutes of Health, Bethesda, MD). Each of the 5 alloys (5 x 3 x 2 mm^3^ cuboids) was evenly mounted into epoxy resin (Epokwick Epoxy resin, Buehler, USA). The epoxy resin was then cut into a long brick (about 45 x 5 x 5 mm^3^).

### Polymer synthesis and coating preparation

Poly (carbonate urethane) urea (PCUU) was synthesized from soft segments of poly (hexamethylene carbonate) diols (PHC, Mn = 2000, Sigma-Aldrich) and 1, 4-diisocynantobutane (Sigma-Aldrich) hard segment with chain extension by 1,4-diaminobutane (Sigma-Aldrich) as previously reported [22, 26]. PHC diols, diisocyanatobutane, and diamine were combined in a 1:2:1 molar ratio [22]. Poly (lactic-co-glycolic acid) (PLGA, L/G = 50:50) with a molecular weight of 30,000–60,000 g/mol was purchased from Sigma-Aldrich. PCUU was dissolved in 1,1,1,6,6,6-hexafluoroisopropanol (HFIP, Oakwood, Inc.) and PLGA was dissolved in chloroform to obtain a 2% (w/v) solution. Polymer coatings were performed by a dip-coating method. Briefly, the alloy-embedded epoxy bricks were dipped into the polymer solution and dried in flowing air for 5 min, which was repeated three times. The specimens were further dried in a fume hood overnight and stored in a vacuum desiccator at room temperature. Polymer-coated and non-coated samples were all sterilized by 70% ethanol incubation for 15 min followed by Milli-Q water immediately prior to the test.

### Microfluidic system design and fabrication

**Fig 1:**
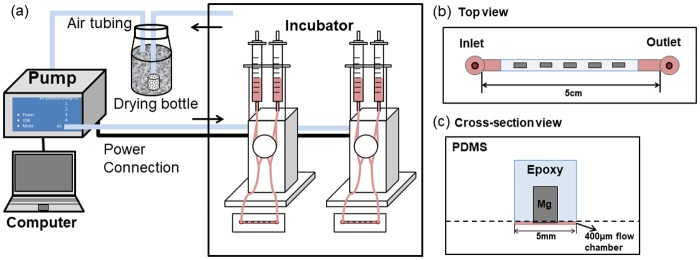
Microfluidic system design. (a) A schematic setup of the microfluidic system composition; (b) Top view and (c) cross-section view of microfluidic channel design.

A microfluidic system was designed to simulate the vascular microenvironment as Fig 1 shows. A pump system (Fig 1(a), ibidi, München, Germany) was used to provide shear stress for standard 0.4 μ-slide flow chamber. The microfluidic chip mold was fabricated according to the protocol of soft-lithography negative photoresist process for SU-8 2100 (Microchem Corp., USA) [27]. The detailed process of wafer chamber fabrication is the same as in the previous publication [3].

PCUU-coated, PLGA-coated, and non-coated alloys (316L SS, WE43, AZ31, ZWEKL and ZWEKC) were embedded into three epoxy bricks. Each epoxy brick had five alloys with the same coating or no coating, and the bricks were attached to the petri dishes with facing to the bottom. Then the polydimethylsiloxane (PDMS; Sylgard 184 Silicone elastomer kit, Dow Corning, Midland, MI, USA) elastomer was poured onto the alloy-embedded epoxy bricks (Fig 1(b)) and negative master wafer, degassed, and allowed to incompletely cure at 60°C overnight. PDMS replicas were pulled off the petri dish and wafer. The two parts of the PDMS were immediately attached together to completely cure with the alloys’ surfaces facing to the channel. A pressure was made to get rid of possible bubbles between PDMS pieces. A 19-gauge blunt-nose needle was used to punch inlet and outlet holes. After assembly with the ibidi pump system, the epoxy-embedded 5 alloys were exposed to the same 400μm thickness chamber (Fig 1(c)) for multi-screening under the same environment.

### Endothelial cell adhesion

The adhesion of endothelial cells on alloy surfaces was tested to evaluate and compare the biocompatibility of endothelial cells with the coated and non-coated alloys. Mice brain endothelial cells (2x10^5/ml) were incubated with completed Dulbecco’s Modified Eagle Medium (DMEM with 10% Fetal Bovine Serum and 1% Penicillin/Streptomycin, ATCC) on alloy surfaces for 4 hours in an incubator (37°C, 5% CO_2_). Then, one set of samples was incubated for 24 hours under static conditions, and the other set of samples was installed in an ibid pump system for dynamic conditions in completed DMEM with shear stress 5 dyne/cm^2^. Each alloy was tested at least three times. A Live/Dead assay kit (Invitrogen Inc.) for mammalian cells was performed to evaluate the cytotoxicity of alloys. The area of live and dead cells (mm^2^) on alloy surfaces was calculated with ImageJ software.

Following static immersion and dynamic experiments, samples were gently rinsed with phosphate buffered saline (PBS, Sigma-Aldrich) 3 times to remove non-adhesive endothelial cells and incubated with glutaraldehyde (2.5% in PBS, Sigma–Aldrich) for 10 min. Next, the materials were washed three times with PBS and dehydrated in consecutive stages of increasing ethanol concentrations. The samples were then chemically dried with hexamethyldisilazane (Thermo Fisher). Finally, the samples were observed using a field emission scanning electron microscope (SEM, SU8000, Hitachi, Japan) after coated with gold-palladium. Element distribution was observed with energy dispersive X-ray spectrometry (EDS, Bruker AXS5350, Germany).

### Pittings study

After a 24-hour test in complete DMEM, samples were washed by acetone and chromic acid solution (200g/L CrO_3_, 10g/L AgNO_3_) to remove the coatings and corrosion products, respectively. Then scanning electron micrographs (SEMs) were obtained after sputter coating.

### Statistical analysis

Statistical analysis (t-test) of endothelial cell adhesion, live/dead cell area (mm^2^), and pitting area (% Area) was performed using Microsoft Excel. The correlation between corrosion rates (mm/year) and pitting areas (% Area) was analyzed with Prism 5. Significance was established at P<0.05. Data are expressed as mean ± standard deviation (SD).

## Results and discussion

### Endothelial cell attachment

Cellular response to biomaterials is an essential factor to evaluate the material biocompatibility [28]. The sequential responses of cells when they contact with a material surface include cell attachment, migration, and proliferation, thus the cells viability of initial response, cell attachment, is an important characterization of alloy compatibility [29]. Endothelial cells are the first cell type of the blood vessel that interacts with the stent during implantation. Researchers have shown the cell attachment on alloy surfaces in static conditions [24, 30, 31]; however, the static attachment test does not reflect the *in vivo* environment, which is subjected to dynamic flow.

**Fig 2:**
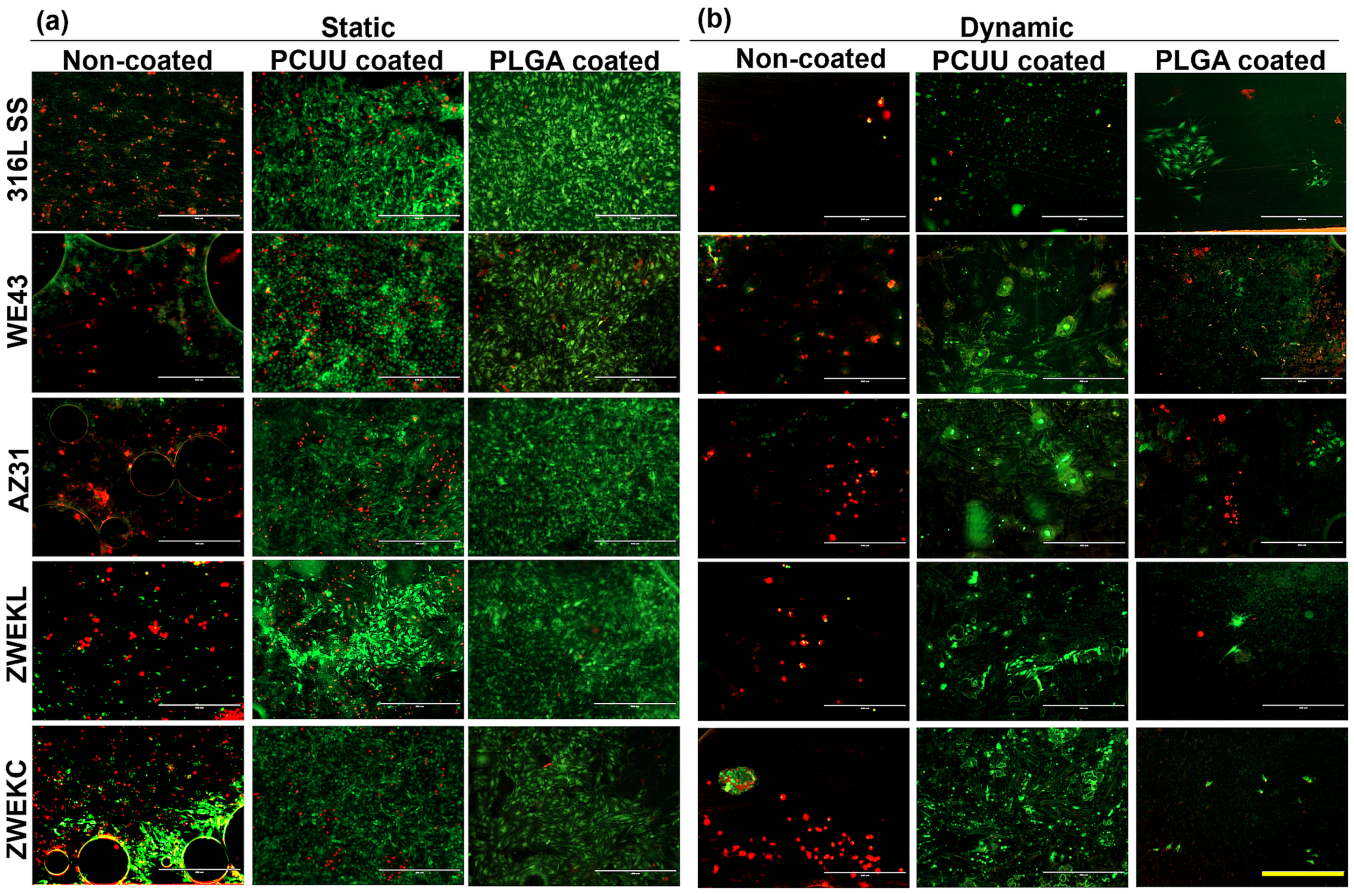
Live/Dead images of endothelial cell. The viability of endothelial cells on Non-coated, PCUU coated and PLGA coated surfaces of 316L SS, WE43, AZ31, ZWEKL and ZWEKC at static (a) and dynamic (b) conditions. Green color represents living cells and red color represents dead cells. Scale bar = 400μm.

**Fig 3:**
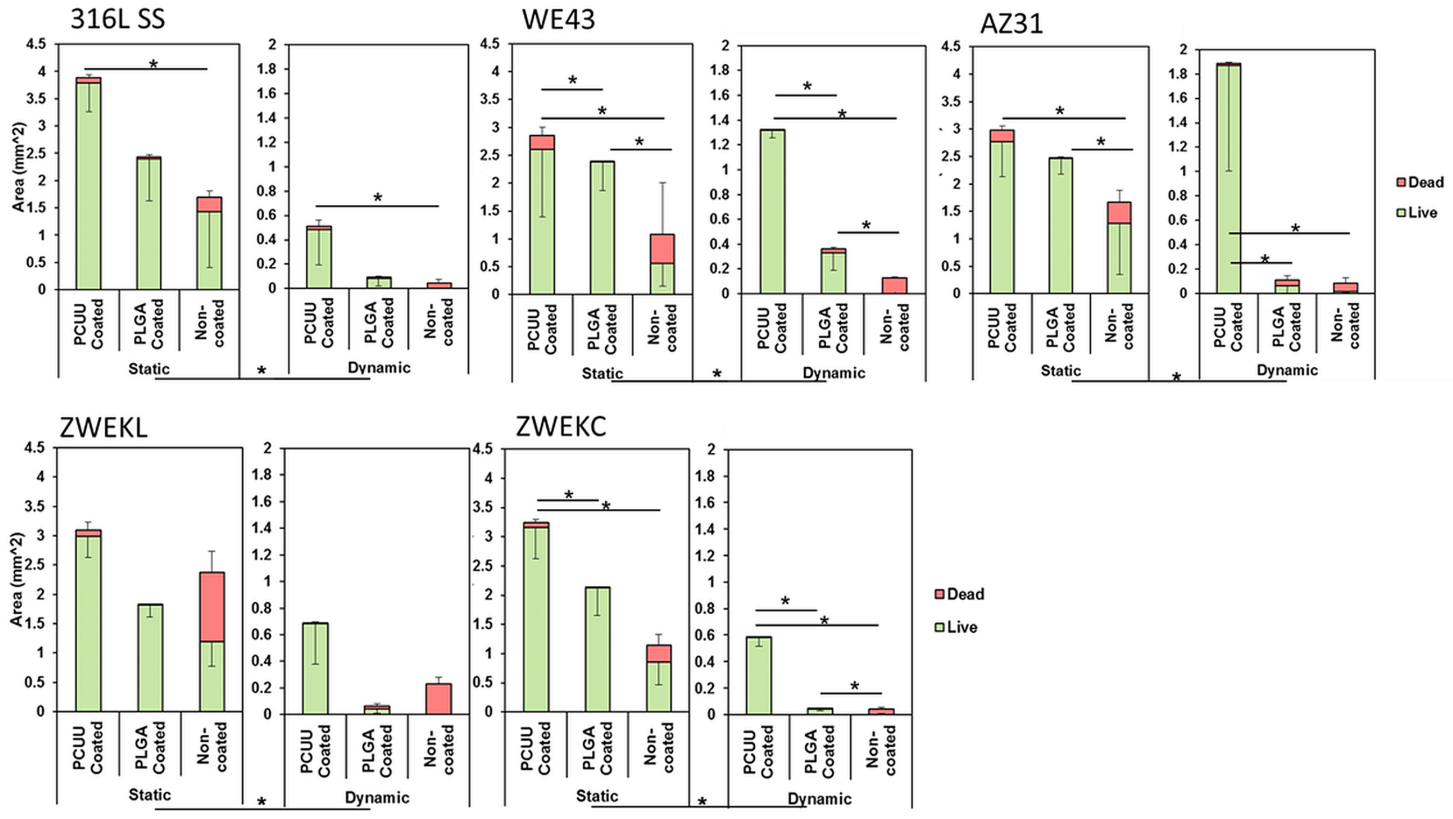
Endothelial cell viability. The green (living cells) and red (dead cells) areas were analyzed by ImageJ software. The quantified bar chart shows areas of living and dead endothelial cells on Non-coated, PCUU coated and PLGA coated surfaces of 316L SS, WE43, AZ31, ZWEKL and ZWEKC at static and dynamic conditions. * represents the significant difference between groups of live/dead ratio.

Here, endothelial cell attachment on samples was tested to evaluate the compatibility of PCUU- and PLGA-coated alloys in both static and dynamic conditions. The viability of attached cells was assessed with live/dead cytotoxicity assays (Fig 2). The green color represents live cells, and the red color represents dead cells. Cells were more viable on the surface of both PCUU- and PLGA-coated alloys than non-coated alloys. The area of live and dead cells was calculated by ImageJ to analyze the viability (Fig 3). The live and dead cell areas (Fig 3) confirmed the observation (Fig 2) that both PLGA and PCUU coatings can improve the viability of endothelial cells. In Fig 3, the viability represented by the live/dead area ratio in static condition is significantly higher than that in dynamic conditions. PCUU coating on the surfaces of 316 L SS, WE43, AZ31, and ZWEKC improved the endothelial cell live/dead ratio in both static and dynamic conditions compared with non-coated alloys (P<0.05). PLGA coating resulted in a higher endothelial cell live/dead ratio on the surfaces of WE43 and AZ31 in static condition, and on WE43 and ZWEKC in dynamic condition compared to non-coated alloys (P<0.05).

The endothelial cell viability on both PCUU-coated and PLGA-coated surfaces of ZWEKL was not improved because the corrosion rate is the highest of all the tested alloys [3], which means a greater amount of hydrogen is released through the coating to repel the cells. The choice of base alloys to be coated is importance. Magnesium alloys with endo-compatibility and re-endothelialization ability *in vitro* and *in vivo* such as Mg-Nd-Zn-Zr (JDBM) are potential choice [32, 33]. PCUU improved live/dead ratios significantly compared with the PLGA-coated surfaces of WE43 and ZWEKC in both static and dynamic conditions, and AZ31 in dynamic condition (P<0.05). PCUU and PLGA improved initial endothelial cell adhesion and PCUU improved endothelial cell viability more than PLGA.

The SEM images of the non-coated and coated alloy surfaces with attached endothelial cells are presented in S2 Fig. In static condition (S2A Fig), cell morphology was normal on PCUU-coated alloys. The cells were integrated with PLGA coatings. At the location of cracks exposing bare alloy surfaces (e.g. Static condition, PCUU coated 316L SS, WE43, and ZWEKL), there are no cells attached. In dynamic condition (S2B Fig), there are cells on PCUU- and PLGA-coated alloy surfaces, but no cells on non-coated alloy surfaces. This indicates that PCUU and PLGA coatings can improve endothelial cell adhesion at the initial stage and PCUU coating has better initial endothelial cell compatibility than PLGA coating. Endothelial injuries can occur during balloon angioplasty, insertion of balloon-expanded stents, or release of self-expanding stents [34]. Since PCUU is elastomeric, it has a more integrated coating on the surface of balloon-expanded stents than PLGA [22]. Long-term endothelial dysfunction happened after stenting in vasculature [35–37]. PCUU coating can be a promising method to improve initial endothelial cell attachment to some alloys, especially those with lower corrosion rates, and facilitate the treatment of endothelial injuries caused by stenting surgery.

### Surface elements distribution

**Fig 4:**
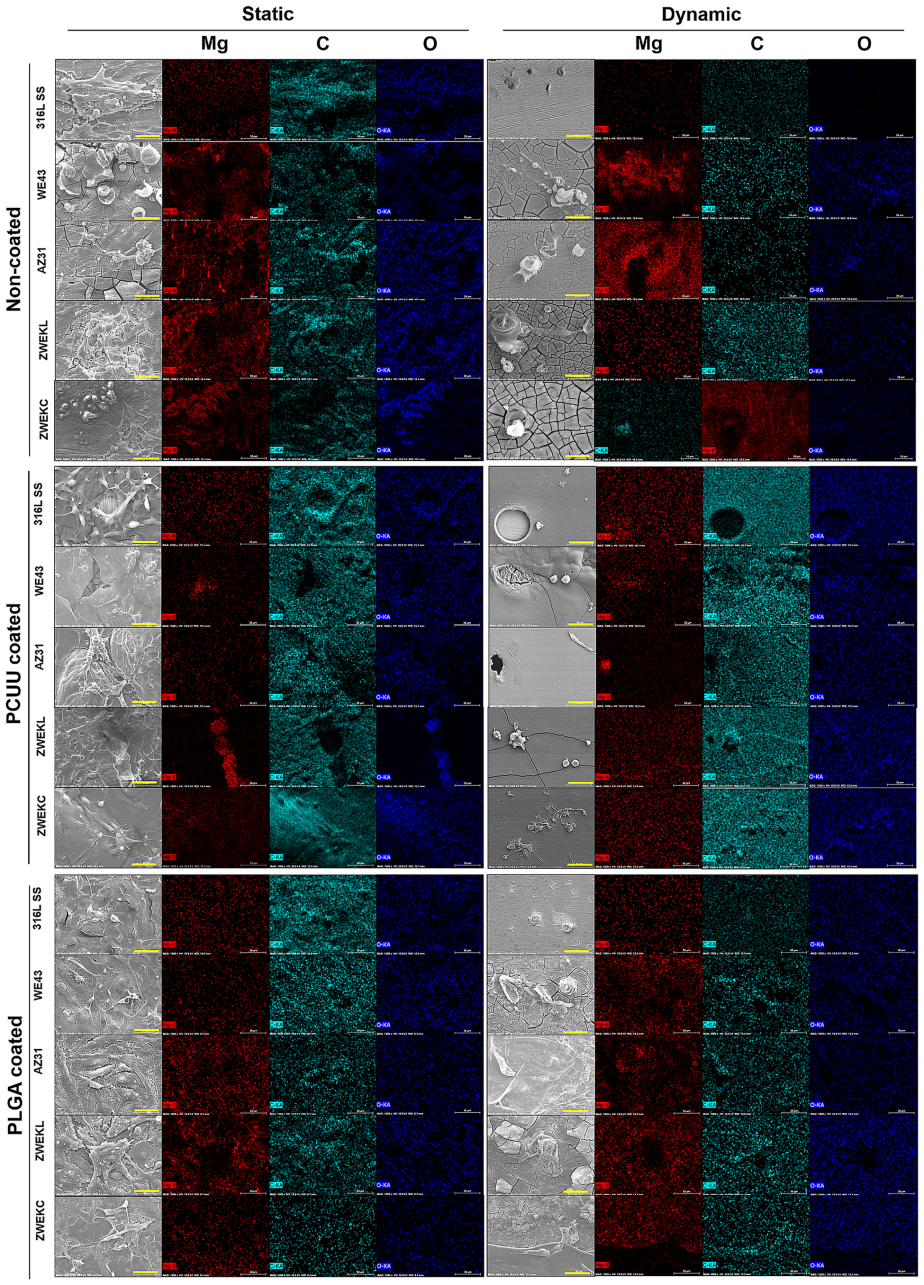
Elementary distribution. After endothelial cell attachment test at static and dynamic conditions, the cells were fixed on Non-coated, PCUU coated and PLGA coated alloys surfaces. Electuary distributions of Mg, C and O on surfaces were scanned using EDS. The light blue accumulation area is carbon rich area, indicating the cells or polymer. The red accumulation area is Mg rich area, indicating the corrosion product or bare Mg-alloys surfaces. This distribution of O is associated with Mg and/or C.

**Fig 5:**
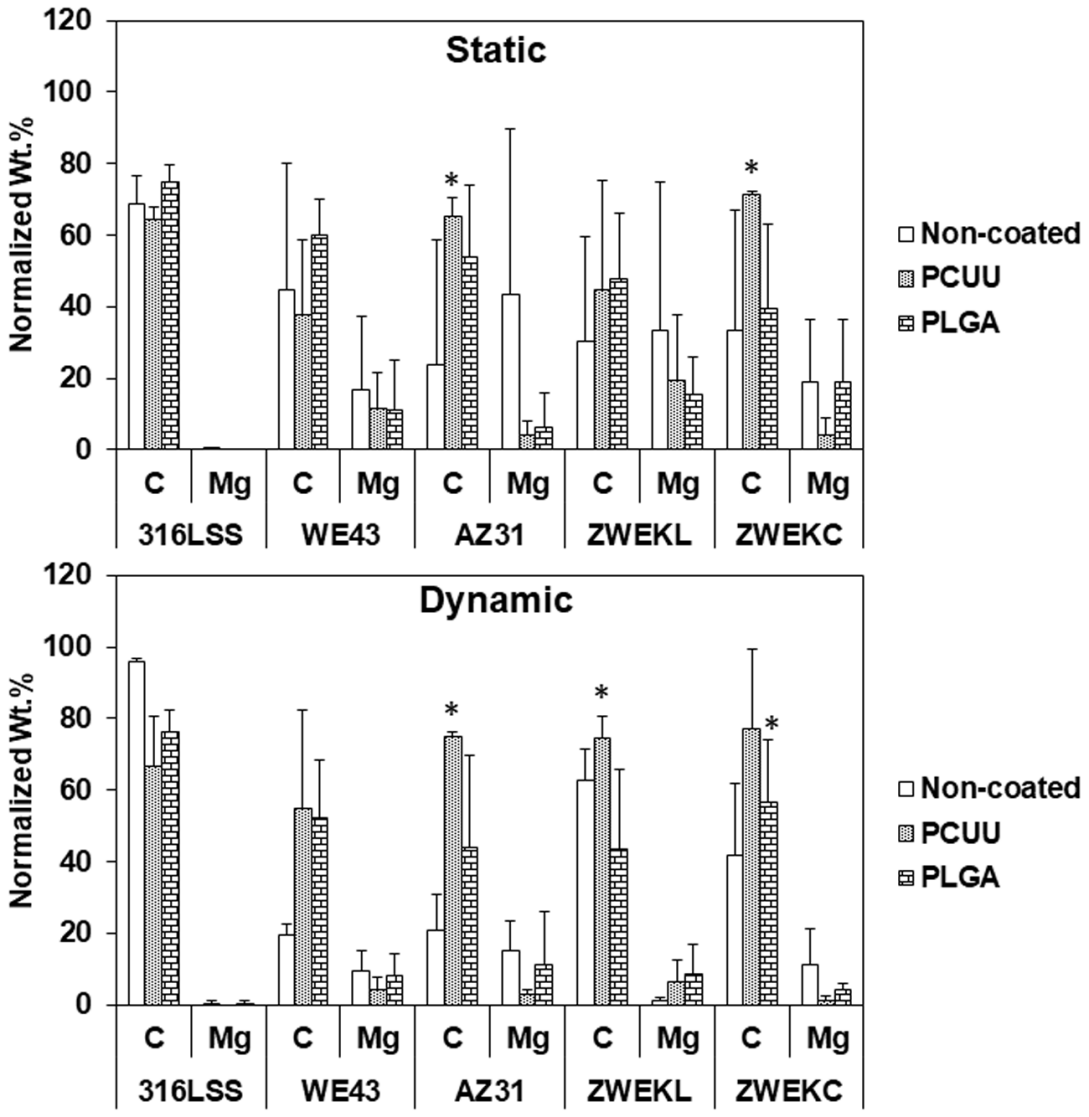
Element weight percentage (Wt%). The normalized Wt% of C and Mg at Non-coated, PCUU and PLGA coated alloys surface after 24 hours’ attachment of endothelial cells. * represents the significant difference between C and Mg for each coated or non-coated alloys (P<0.05).

Carbon (C), magnesium (Mg), oxygen (O), calcium (Ca), and phosphorus (P) were analyzed with EDS. The overall distribution mapping is shown in Fig 4. EDS point mapping was performed on three points (S3 Fig). The coated surfaces after the endothelial cell attachment test in both static and dynamic conditions have complex elemental compositions and distributions. As a control sample, the 316L SS surface mainly consists of iron and no magnesium. In the results, iron was eliminated in mapping for analysis. There are four basic hypothesized substances on Mg alloy surfaces: cells, coating, corrosion products, and bare alloy surfaces. Cells and coatings are carbon-rich. The Mg weight percentage is generally higher in static condition compared to dynamic condition (Fig 5). The reason is that in dynamic condition, the released Mg ions are taken away from the alloy surfaces by dynamic flow. The carbon weight percentages are all higher than Mg, and significantly higher on PCUU-coated AZ31 in both static and dynamic conditions, PCUU-coated ZWEKL in dynamic conditions, PCUU-coated ZWEKC in static conditions, and PLGA-coated ZWEKC in dynamic conditions (P<0.05). Generally, the PCUU-coated alloys (AZ31 and ZWEKC) have higher carbon and lower Mg. This indicates PCUU-coated AZ31 and ZWEKC have more cells and/or coatings than PLGA. PCUU can be a better coating than PLGA on AZ31 and ZWEKC.

The released Mg ions on the surface indicate exposure of bare Mg alloy surfaces and/or corrosion products after coating degradation. More carbon means less corrosion, more cells, or larger amount of coating, which indicates either the coating improved endothelial cell attachment or had plying-up quality on alloy surfaces. This is the expected situation, which can facilitate screening the best-fit polymer coating for a specific alloy surface under specific conditions. A higher Mg weight percentage means greater Mg ion release, which means more corrosion. As a corrosion product, Mg ions were released from corroded positions and diffused to the surface of the coating and cells. Some standard deviations of carbon and Mg are large because of the uneven distribution of cells, coating, and corroded positions.

### Pittings

**Fig 6:**
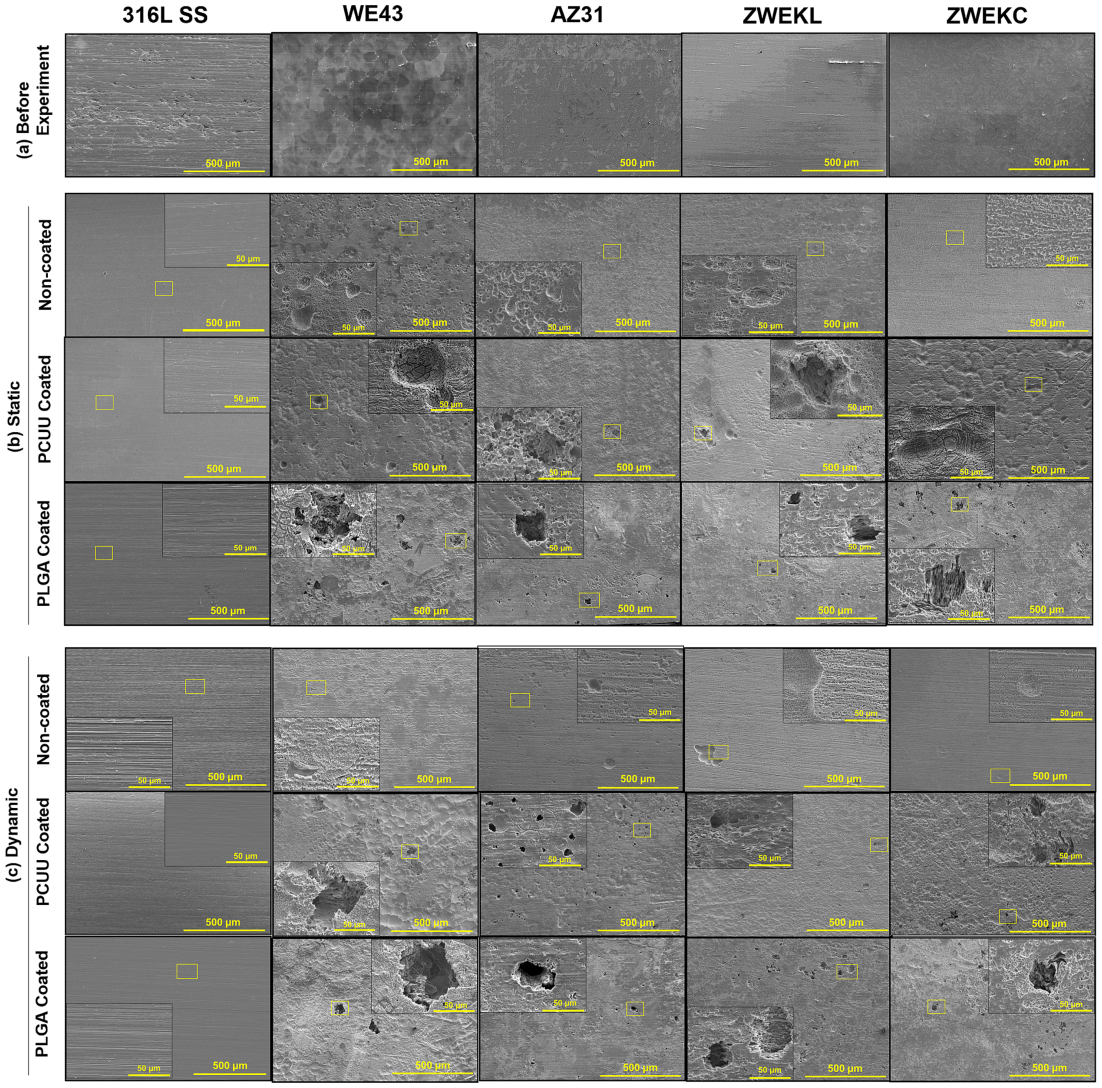
Pittings morphologies. There is no pittings on surfaces of alloys before experiment (a). After 24 hours’ endothelial attachment test at static (b) and dynamic (c) conditions, attached endothelial cells, corrosion product and left coatings were removed. Then the surfaces of alloys were observed under SEM and various sizes of pittings were found on alloys surfaces. Scale bar = 500μm and 50μm.

**Fig 7:**
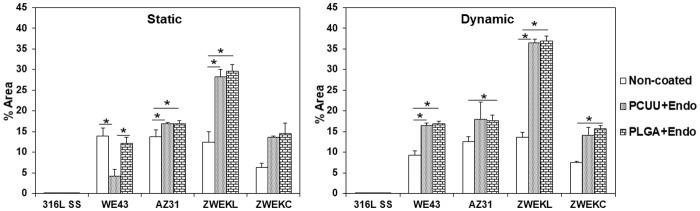
Pitting areas percentage (% Area) on alloys’ surfaces tested in static and dynamic conditions. The pittings were observed by SEM on surfaces of Non-coated, PCUU, PLGA coated alloys after removing attached endothelial cells, left coating and corrosion products. The pitting areas and percentage on alloys surfaces and under coatings were calculated with ImageJ software. * represents the significant difference of between pitting area percentages of Non-coated, PCUU and PLGA coated alloys on same alloy’s surface (P<0.05).

Cells and coatings were removed to observe alloy surfaces by SEM. There were pittings of various sizes underneath both PCUU and PLGA coatings on Mg alloys (Fig 6). The pitting areas were calculated by ImageJ (Fig 7). In static condition, PCUU-coated WE43 had significantly less pitting areas compared to non-coated and PLGA-coated alloy surfaces (P<0.05); PCUU- and PLGA-coated AZ31, PCUU- and PLGA-coated ZWEKL had significantly more pitting areas compared to non-coated alloy surfaces (P<0.05). In dynamic condition, PCUU- and PLGA-coated WE43 and ZWEKL, PLGA-coated AZ31 and ZWEKC had more pitting areas compared to non-coated alloys (P<0.05). In our previous study, we found ZWEKL had the most rapid degradation of all the tested alloys [3], and in this study we found that it has the most severe pittings underneath the coating.

It has been shown that both PLGA and PCUU coatings can be used to provide corrosion resistance to alloys [22, 24]. The pittings showed us that even if PCUU and PLGA coating can protect Mg alloys from degrading too rapidly, there is still a chance that pittings will occur on alloy surfaces. The correlation of pittings and corrosion rates was analyzed with Prism 5 (S4 Fig). All pitting area percentages of whole surfaces had positive but not significant correlation with corrosion rates in dynamic conditions, because WE43, AZ31 and ZWEKC have similar and lower corrosion rates than ZWEKL.

The pittings on biodegradable Mg alloy surfaces occur due to the penetration of polymeric coatings upon degradation [24]. Once the coating begins to degrade at some positions, the fresh reactive surface underneath is exposed to the corrosive medium, which accelerates degradation. The pittings on alloy surfaces are more severe when there are coating defects. Defects in the coating are likely to occur where hydrogen gas is rapidly generated, leading to pitting corrosion on the Mg substrates. The coatings prevented hydrogen gas from being released to the medium, causing the formation of balloon-like structures on alloy surfaces (e.g. S2A Fig, static condition, PCUU-coated AZ31). The trapped hydrogen gas would then lead to hydrogen absorption and subsequent embrittlement of alloys [38], causing rapid uneven corrosion of Mg alloys. Pittings can induce hydrogen embrittlement of Mg alloys, leading to alloy damage [39]. When the amount of hydrogen gas generated was too great, the coating would break, leaving a hole on the surface of PCUU-coated AZ31 in dynamic condition (S2B Fig). Crevice corrosion and increased localized pH are other possible reasons for pitting. Crevice corrosion occurs in confined spaces between the coating and Mg alloy surface, to which the access of the medium from the environment is limited. Degradation generates OH^-^, which is not immediately balanced or buffered by the medium due to the separation by the coating. This causes increased localized pH at polymer-coated surfaces, thus leading to localized pittings. This kind of pitting can be reduced by combining the polymeric coatings with other effective methods, such as the micro-arc technique (MAO) [40, 41].

## Conclusion

The test of endothelial cell attachment indicates that both PCUU and PLGA coatings on Mg alloys are capable of improving initial endothelial cell adhesion. PCUU coating has a greater ability to improve endothelial cell attachment on more alloy surfaces compared to PLGA coating. Pittings are more severe on the surface of Mg alloys that corrode rapidly, thus coatings are strongly recommended be applied on Mg alloys with relatively low corrosion rates, or on pre-stabilized Mg substrates.

## Supporting information

S1 Fig Microstructure information of Mg-based alloys. A, Grain structure of magnesium-based alloys: WE43, AZ31, ZWEKL and ZWEKC. Grain diameter (D) are: D (WE43) = 105.5±35.1μm, D (AZ31) = 16.5±9.1μm, D (ZWEKL) = 5.0±1.4μm, D (ZWEKC) = 5.4±1.6 μm. B, the secondary phases and impurity area percentage (% Area).
(TIF)

S2 Fig Endothelial cell morphologies and corrosion morphologies. After 24 hours’ endothelial cell attachment test, the surfaces of Non-coated, PCUU coated and PLGA coated alloys were observed under SEM. A, Endothelial cell attachment at static condition; B, Endothelial cell attachment at dynamic condition.
(TIF)

S3 Fig Spectrum acquisition of selected three positions (1, 2, and 3). Weigh percentage (Wt.%) of C, Mg, O, Ca, P on Non-coated (a), PCUU coated (b) and PLGA coated (c) surfaces with EDS. Scale bar = 30μm.
(TIF)

S4 Fig Correlation between corrosion rate and pitting area underneath coating in both static and dynamic conditions. (TIF)
