# Supplementary figures and images for: Comparison of endothelial cell attachment on surfaces of biodegradable polymer-coated magnesium alloys in a microfluidic environment
Source: PLoS One. 2018 Oct 10;13(10):e0205611. doi: 10.1371/journal.pone.0205611 (PMC6179289; doi:10.1371/journal.pone.0205611)

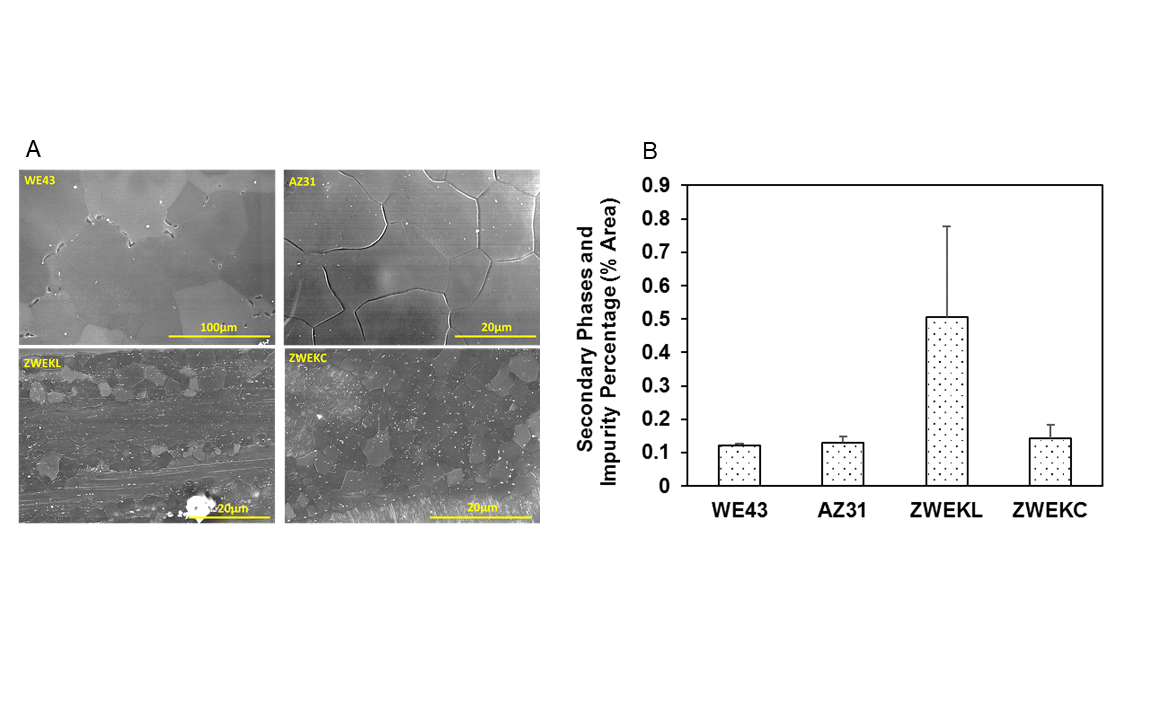

Supplement: S1 Fig — A, Grain structure of magnesium-based alloys: WE43, AZ31, ZWEKL and ZWEKC. Grain diameter (D) are: D (WE43) = 105.5±35.1μm, D (AZ31) = 16.5±9.1μm, D (ZWEKL) = 5.0±1.4μm, D (ZWEKC) = 5.4±1.6 μm. B, the secondary phases and impurity area percentage (% Area). (TIF) [file pone.0205611.s001.tif]

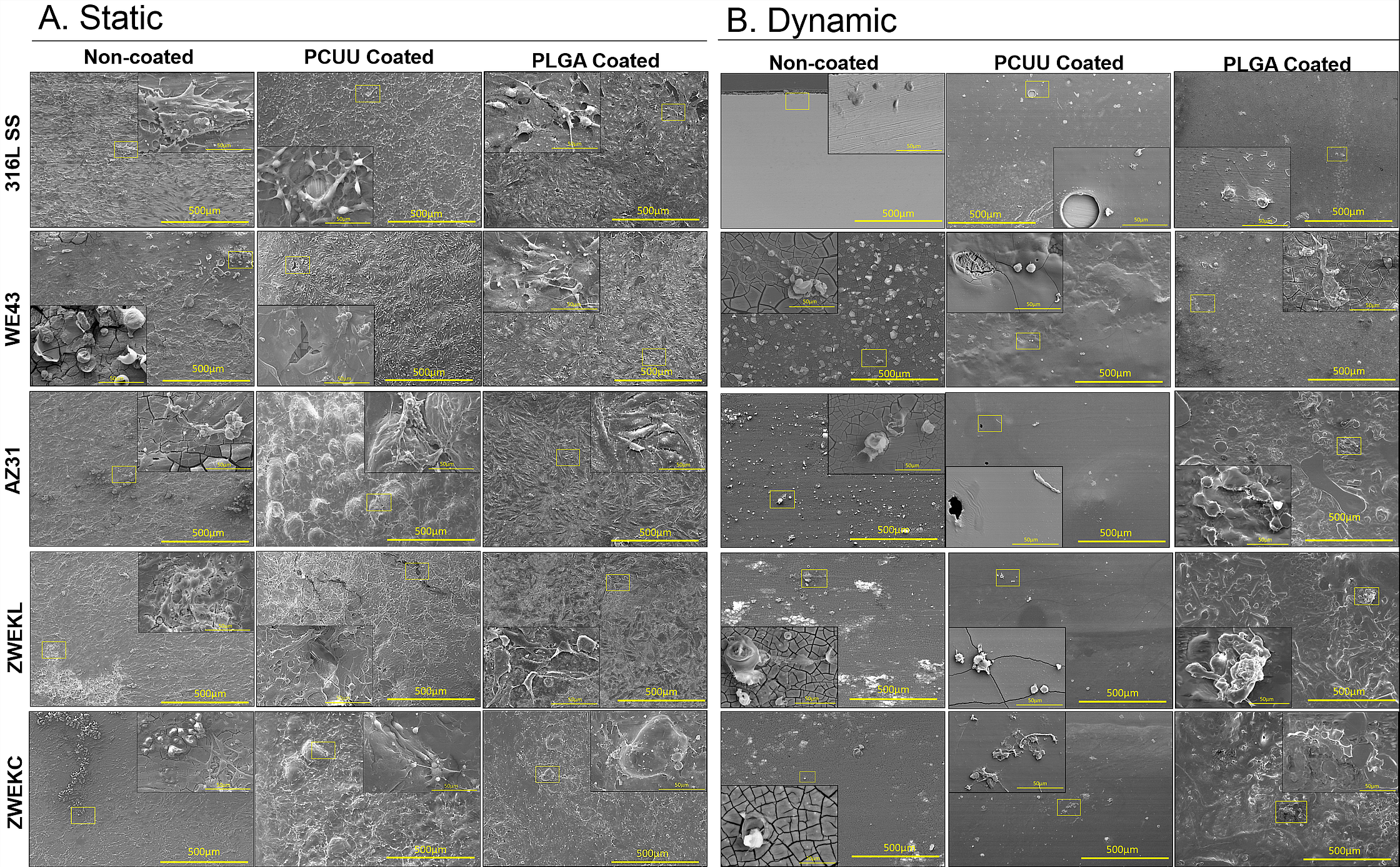

Supplement: S2 Fig — After 24 hours’ endothelial cell attachment test, the surfaces of Non-coated, PCUU coated and PLGA coated alloys were observed under SEM. A, Endothelial cell attachment at static condition; B, Endothelial cell attachment at dynamic condition. (TIF) [file pone.0205611.s002.tif]

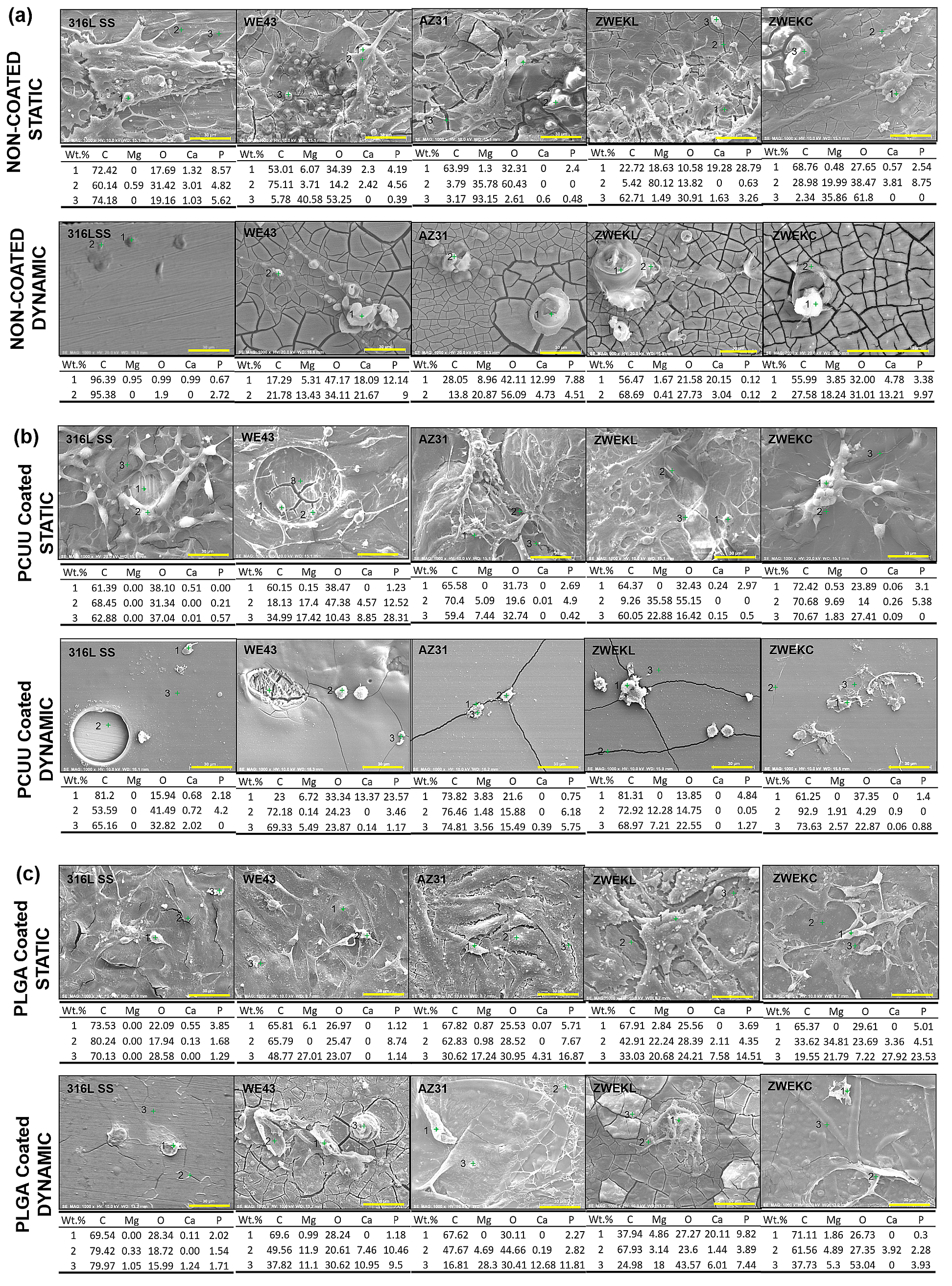

Supplement: S3 Fig — Weigh percentage (Wt.%) of C, Mg, O, Ca, P on Non-coated (a), PCUU coated (b) and PLGA coated (c) surfaces with EDS. Scale bar = 30μm. (TIF) [file pone.0205611.s003.tif]

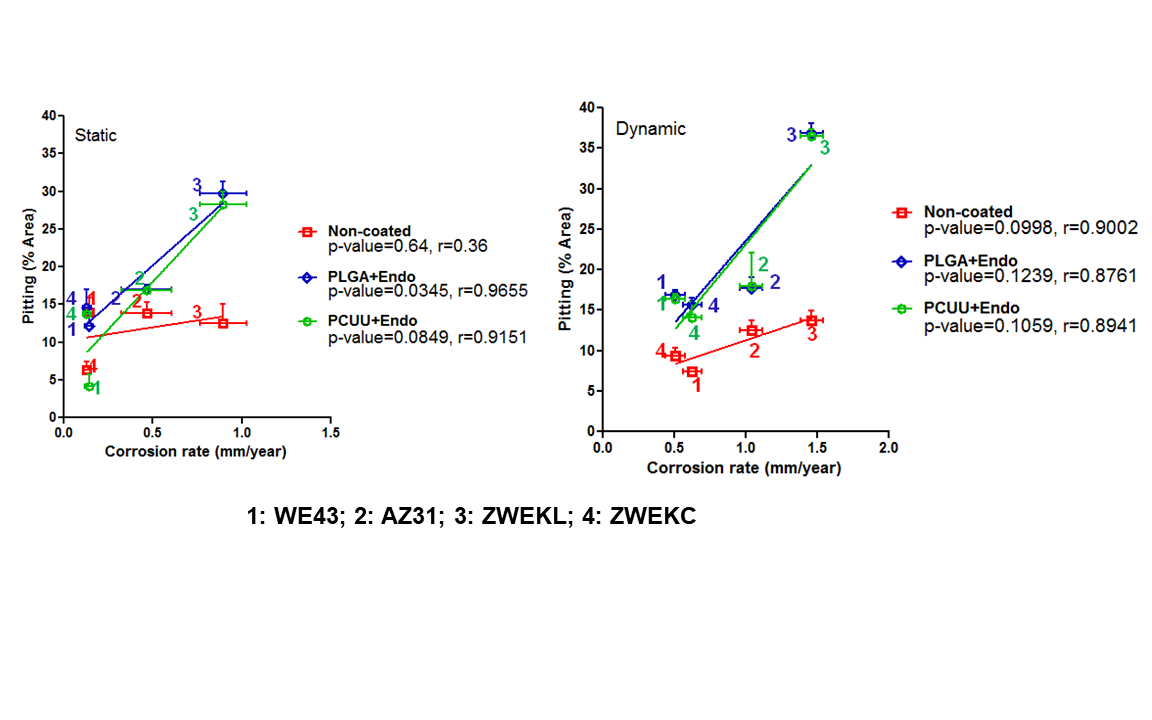

Supplement: S4 Fig — (TIF) [file pone.0205611.s004.tif]
